# Examining healthcare staff views and experiences with equity, diversity, and inclusion (EDI) in a multi-disciplinary healthcare setting: A mixed methods needs assessment to advance inclusive excellence

**DOI:** 10.1177/09514848251387042

**Published:** 2025-10-09

**Authors:** Lauren R Squires, Logan Meyers, Myann Marks, Eryn Tong, Ekaterina An, Camilla Zimmermann, Jacqueline L Bender

**Affiliations:** 1 Department of Supportive Care, Princess Margaret Cancer Centre, 7989 University Health Network, Toronto, ON, Canada; 2 Dalla Lana School of Public Health, University of Toronto, Toronto, ON, Canada

**Keywords:** equity, diversity, inclusion, EDI, healthcare, workplace EDI

## Abstract

Equity, diversity, and inclusion (EDI) is increasingly identified as a priority in healthcare organizations and an essential component of high-quality care. However, research on advancing EDI in healthcare workplaces is limited. This study sought to elucidate how to advance inclusive excellence in a clinical department of a comprehensive cancer centre. A mixed-methods quality improvement project was undertaken whereby staff completed an online survey, and a sub-group were interviewed. Quantitative data were summarized using descriptive statistics and univariate regression analyses and qualitative data were analyzed using thematic analysis. 103 of 219 staff/learners completed the survey and 17 staff were interviewed. Over 90% of survey participants agreed EDI should be a priority and 29% had experienced discrimination, which was associated with considering leaving the organization. Facilitators to EDI were: enthusiasm/awareness of EDI, openness to new ideas, gender diversity, and safe environments for self-expression. Barriers to EDI were lack of: EDI knowledge, cohesion/collaboration, psychological safety, diversity along various dimensions, EDI-related communication, and burnout. To advance departmental EDI, initiatives should leverage facilitators and overcome barriers to meet department needs aligning with organizational goals. These findings will inform the development of a story huddle learning series to strengthen EDI-related knowledge and skills.

## Background

Workplace equity, diversity, and inclusion (EDI) initiatives aim to promote fairness, address systemic oppression, encourage staff diversity, and create an inclusive environment that fosters belonging where all staff can thrive regardless of characteristics such as race, age, gender, sexual orientation, income, nationality, or religious affiliation.^1^ Mainstream focus around EDI has seen a dramatic increase in North America within the past 5 years, due in no small part to the upsurge in awareness of various social justice movements (e.g., Black Lives Matter) and the onset of the COVID-19 pandemic.^2^ In response, organizations have concentrated time and effort on improving workplace EDI, including implementing EDI initiatives and diversifying hiring practices.^1^ Healthcare is no exception, where EDI is increasingly identified as a strategic priority and an essential component of high-quality person-centred research and care.^1,3^

### Workplace EDI needs assessments

While there are examples of workplace EDI initiatives in the literature,^1^ there are few published reports of needs assessments undertaken by organizations to inform the development of EDI initiatives. Needs assessments are a critical part of EDI advancement, as they allow organizations to assess and reflect on their EDI status, develop initiatives tailored for their members, and track progress toward their EDI goals. A foundational example of an industry EDI initiative informed by organizational needs is the diversity initiative undertaken by former IBM CEO Lou Gerstner in 1995, who created a task force of employees to better understand the company’s diversity needs and concerns.^4^ The result was increased company revenue and better recruitment and retention of diverse employees achieved through four pillars of change: “demonstrat[ing] leadership support; engag[ing] employees as partners; integrat[ing] diversity with management practices; and link[ing] diversity goals to business goals” (pp. 104-107).^4^ An example in higher education is the EDI strategy of McMaster University in Canada. Their EDI strategy, highlighting the relevance of EDI at every level and emphasizing the role of collective responsibility in EDI capacity-building, was developed following extensive consultation sessions with various stakeholders (e.g., students, faculty/staff, senior leaders).^5^ Stakeholder feedback informed the final EDI framework and strategy, thereby ensuring collective responsibility for EDI advancement at all institutional levels. In conducting structured needs assessments before implementing EDI initiatives, both organizations demonstrated a commitment to inclusive excellence that emphasized *collective ownership* over EDI advancement by linking inclusive excellence with overall productivity and success.

### EDI in the healthcare workplace

The central focus of all healthcare organizations is providing equitable, quality care to achieve optimal health outcomes, of which ensuring patients are treated by diverse healthcare teams is a critical component.^6^ The benefits of diverse providers cannot be overstated, particularly for equity-seeking groups who may feel greater safety with providers who share similar identity characteristics.^6^ By extension, healthcare organizations are responsible for recruiting and retaining a diverse workforce to ensure optimal patient care. However, many providers from equity-seeking groups still experience discrimination and exclusion at work due in part to a culture that values standards of medical professionalism privileging Western, white, cisgender, heterosexual men.^7^

Such experiences and fear of being seen as unprofessional can contribute to providers from equity-seeking groups working harder than their non-equity-seeking counterparts to appear professional, speak up about the importance of EDI, and take on mentorship responsibilities over and above patient care responsibilities.^7^ While factors such as gender or racial/ethnic background certainly exacerbate power hierarchies within healthcare, institution-specific factors such as profession (e.g., physician, nurse, allied health professional) and position (e.g., leadership, middle management) can heavily influence hierarchical position.^8^ Predictably, these structures can make it more difficult for people to speak up at work and address discriminatory and exclusionary identity-based hierarchies.

There is a notable gap in the literature regarding the EDI-related needs and experiences of staff in healthcare organizations. Most articles that have explored the EDI-related needs and experiences of healthcare staff were conducted through professional associations, meaning participants were employed at different institutions across geographic locations.^9,10^ To our knowledge, a New Zealand study by Kuntz and Pandaram is the only prior study to examine the EDI-related perceptions of employees at a single healthcare organization.^11^ They explored the relationship between employees’ sense of belonging and their perceptions of the workplace EDI climate, and assessed differences in belonging between ethnic minority and majority staff. They found that ethnic minority employees felt a lower sense of belonging than their majority counterparts, but that there was congruence between organizational EDI commitment and perceived belonging among staff. More research is needed to understand the EDI-related needs of healthcare staff in different settings and regions of the globe to *advance* workplace EDI in areas where it is lacking.

### The current study

To address this gap and develop contextually-relevant EDI initiatives, an EDI needs assessment was undertaken in the Department of Supportive Care (DSC) in the University Health Network (UHN) in Toronto, Canada. The DSC is interdisciplinary, employing approximately 200 physicians, allied health professionals, and researchers focused on psychosocial research and care. Most of the department’s services (90%) are provided to people affected by cancer and their families, and the remainder (10%) to those affected by other conditions. The needs assessment followed the creation of two EDI co-lead roles to reflect the department’s commitment to inclusive excellence. The overarching objective was to better understand staff/learner perspectives on and experiences with departmental EDI, with the goal of developing tailored EDI initiatives.

## Methods

### Study design

An explanatory sequential mixed-methods design was used,^12^ involving an anonymous online survey distributed to all department staff and learners and semi-structured interviews with a purposive sub-group. The survey and interview data were integrated to provide a comprehensive summary and explanation of the findings.^12^ This study was reviewed and approved by the UHN Quality Improvement Institutional Review Board (QI ID: 22-0479), which reviews all studies using quality improvement methods, including those for peer-reviewed publication. All participants were informed in writing about the purpose of the study, what participation involved, and how the data would be protected and used. We followed the Good Reporting of A Mixed Methods Study (GRAMMS) guidelines^13^ in the reporting of the results (see Supplemental Appendix 1).

### Survey

#### Recruitment

An anonymous and confidential survey was distributed to all department staff (including administrative, clinical, and research) and learners (including clinical and research) from April 2022 to April 2023. The survey was hosted online using REDCap and was distributed by email using departmental distribution lists. Two follow-up reminder emails were sent 1 week apart to encourage survey completion. To incentivize participation, respondents were invited to provide their contact information to receive a $5 Starbucks gift card upon completion. To protect anonymity, participants’ contact information was not linked to the survey.

#### Survey development

The survey was developed based on an environmental scan of diversity and climate surveys conducted by healthcare institutions, universities, and industries in Canada and the United States. It was pilot tested with three department staff before distribution and included five sections: (1) demographics; (2) EDI knowledge; (3) EDI climate; (4) experiences with micro-aggressions, discrimination, and harassment; and (5) initiatives to advance inclusive excellence. Demographic questions were adapted from the SPARK Demographic Data Collection Tool.^14^ Questions on EDI climate, microaggressions, discrimination/harassment, and initiatives to advance inclusive excellence were adapted from the McMaster Diversity and Climate survey^15^ with select questions from the Harvard Diversity and Climate survey.^16^ The Gartner Inclusion Index was used to measure seven dimensions of inclusion: fair treatment, integrating differences, decision-making, psychological safety, trust, and diversity.^17^ Questions were presented on a 7-point Likert scale (e.g., 1 – Strongly disagree to 7 – Strongly agree). See Supplemental Appendix 2 for the full survey.

#### Analysis of survey data

Data analysis was conducted in SPSS version 28. For continuous variables, mean, standard deviation, median, first quartile (Q1), third quartile (Q3), minimum and maximum were calculated. For categorical variables, frequency and percentage were presented. Univariate logistic regression analysis was conducted to investigate associations between participant sociodemographic factors (country of birth, race, gender, sexual orientation, religion, disability, age, and position within the department dichotomized on the basis of equity-/non-equity-seeking groups), considering leaving the department (dichotomized into yes/no), and experiencing micro-aggressions, discrimination or harassment in the organization (dichotomized into yes/no).

### Staff interviews

#### Recruitment and data collection

Formative semi-structured interviews were conducted with department staff to understand their perceptions of EDI climate and barriers and facilitators to addressing departmental EDI needs. A purposive maximum variation sample^18^ was sought with representation from all department sites and teams, both clinical and research, across three categories of staff (leadership, research/program coordination, and administration). Interviews were approximately 60 min and followed an interview guide developed by the EDI team. Audio-recorded interviews were conducted jointly by MM and JLB and were transcribed verbatim.

#### Analysis of interview data

Data were analyzed following Braun and Clarke’s^19^ procedure for thematic analysis using NVivo version 12. After completing the first step of familiarizing themselves with the data, EA, ET, JLB, and LS generated initial codes of two transcripts independently. After coding the initial two transcripts, EA, ET, JLB, and LS met to compare codes and reach consensus. EA and LS then created a codebook to guide independent coding of the remaining transcripts, which was followed by the third and fourth steps of generating and reviewing themes. Final themes were then defined and named to prepare the final report.^19^ Throughout analysis and development of themes, LS regularly met with the EDI project team to discuss the findings.

#### Data integration

Quantitative and qualitative data were analyzed separately. Qualitative data were then used to contextualize nuances in the quantitative data in narrative format, facilitating integration and interpretation. Data integration was undertaken primarily by LS, with input from co-authors.

## Results

### Department survey

A total of 219 departmental members were sent a survey invitation, and 103 surveys were completed (47% response rate). Of the 103 participants, 88% were staff and 11% learners (Table 1). Approximately two-thirds of survey participants were born in Canada (62%) and identified as white (57%). Nearly half were aged 30-49 (46%) and 41% were responsible for dependents (e.g., a child, parent, or other family member/relative, person with a disability, etc.). Most survey participants identified as women (87%), heterosexual (81%), and reported no disability (80%). A majority (81%) of survey participants’ parents’ highest level of education was a university degree.

**Table 1.** Survey participant characteristics.

Participant characteristic	n (%)
Place of birth (n = 98)	
Canada	67 (62)
Other	30 (27.8)
Prefer not to answer	1 (0.9)
Age (n = 101)	
20-29 years old	21 (19.4)
30-49 years old	50 (46.3)
50-70 years old	25 (23.1)
Prefer not to answer	5 (4.6)
Gender (n = 103)	
Woman	90 (87.4)
Man	9 (8.7)
Prefer not to answer	4 (3.9)
Sexual orientation (n = 103)	
Heterosexual	83 (80.6)
Sexually diverse	15 (14.6)
Do not know	1 (1)
Prefer not to answer	4 (3.9)
Indigenous status (n = 103)	
Yes	1 (1)
No	100 (97.1)
Prefer not to answer	2 (1.9)
Race (n = 103)	
White European/North American	59 (57.3)
Racialized	40 (38.8)
Prefer not to answer	4 (3.9)
Religion (n = 103)	
Christian/Christian Orthodox/Protestant/Roman Catholic	33 (32)
Other	66 (64.1)
Prefer not to answer	4 (3.9)
Disability (n = 103)	
Yes	19 (18.5)
No	82 (79.6)
Do not know	1 (1)
Prefer not to answer	1 (1)
Disability accommodation (n = 103)	
Yes	5 (4.9)
No	20 (19.4)
Do not require	77 (74.8)
Prefer not to answer	1 (1)
Care for dependents (n = 101)	
Yes, full-time	28 (25.9)
Yes, part-time	16 (14.8)
No	55 (50.9)
Prefer not to answer	2 (1.9)
Parent education (n = 101)	
Graduated high school or less	5 (4.6)
Attended college/CEGEP/university	2 (1.9)
Completed college/skilled trade degree	10 (9.3)
Completed university degree	82 (81.2)
Prefer not to answer	2 (1.9)
Position in the department (n = 99)	
Staff	87 (87.9)
Learner	11 (11.1)
Prefer not to answer	1 (1)

#### EDI knowledge and learning stage

Over 90% of survey participants agreed that EDI should be a leading priority of the department, and 83% agreed they had working knowledge of unconscious bias (Table 2). Less agreed that they had working knowledge of anti-oppression (60%) and cultural safety (57%). Nearly three-quarters (71%) agreed that they regularly apply EDI principles to their work, learning, or teaching.

**Table 2.** EDI-related knowledge.

Self-reported EDI Knowledge	n (%)
1. I believe EDI should be a leading priority for the department. (n = 96)	
Strongly disagree/disagree	0 (0)
Somewhat disagree/neither agree nor disagree/somewhat agree	7 (7.3)
Strongly agree/agree	88 (91.7)
Prefer not to answer	1 (1)
Missing	**7**
2. I have working knowledge of unconscious bias. (n = 96)	
Strongly disagree/disagree	0 (0)
Somewhat disagree/neither agree nor disagree/somewhat agree	16 (16.7)
Strongly agree/agree	79 (82.3)
Prefer not to answer	1 (1)
Missing	**7**
3. I regularly apply EDI principles in my work/teaching/learning. (n = 96)	
Strongly disagree/disagree	0 (0)
Somewhat disagree/neither agree nor disagree/somewhat agree	27 (28.1)
Strongly agree/agree	68 (70.8)
Prefer not to answer	1 (1)
Missing	**7**
4. I have working knowledge of anti-oppression. (n = 96)	
Strongly disagree/disagree	1 (1)
Somewhat disagree/neither agree nor disagree/somewhat agree	37 (38.5)
Strongly agree/agree	57 (59.4)
Prefer not to answer	1 (1)
Missing	**7**
5. I have working knowledge of cultural safety. (n = 96)	
Strongly disagree/disagree	1 (1)
Somewhat disagree/neither agree nor disagree/somewhat agree	40 (41.7)
Strongly agree/agree	54 (56.3)
Prefer not to answer	1 (1)
Missing	**7**

**Figure 1.**
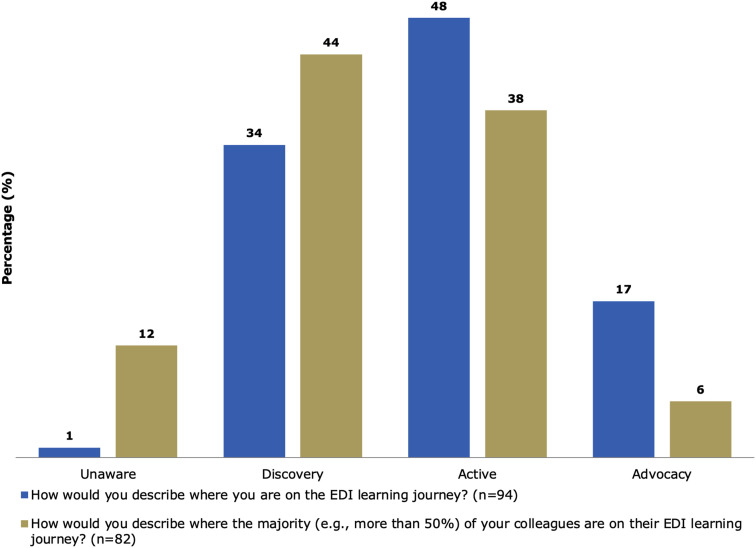
Perceived EDI learning journey of self and department colleagues. Unaware – Unaware of the impact EDI can have on people and work. Discovery – Interested in learning about the issues and barriers that stand in the way of EDI. Active – Advancing knowledge, engaging in tough conversations, and actively applying EDI principles in their work. Advocacy – Championing the advancement and success of others including those marginalized by gender, Indigenous Peoples, racialized minorities, persons with disabilities, and members of 2SLGBTQ + communities.

When asked where they believed they were on their own EDI learning journey (i.e., unaware, discovery, actively working to advance EDI, or advocating and championing for EDI), nearly half (48%) of survey participants believed they were in the active stage (Figure 1). When asked to reflect on where they thought most of their colleagues were on their EDI learning journey, most (44%) felt their colleagues were in the discovery stage.

#### Workplace inclusion

Over half of survey participants agreed that ideas and suggestions are fairly considered in decision-making (fair decision-making; 70%), that people in the department respect and value each other’s opinions (integrating differences; 64%), that communication from department administration and leadership is open and honest (trust; 63%), and that they felt cared for by colleagues (belonging; 55%; Table 3). Less than half agreed that they felt welcome to express their true feelings at work (psychological safety; 47%), that people in the department were rewarded and recognized fairly (fair treatment; 38%), and that department managers are as diverse as the broader workforce (diversity; 28%).

**Table 3.** Perceived departmental EDI climate.

Inclusion index	n (%)
1. Members of my team fairly consider ideas and suggestions offered by other team members. (n = 91)	
Strongly disagree/disagree	1 (0.97)
Somewhat disagree/neither agree nor disagree/somewhat agree	26 (25.2)
Strongly agree/agree	63 (61.2)
Prefer not to answer	1 (0.97)
Missing	**12**
2. People in the department respect and value each other’s opinions. (n = 91)	
Strongly disagree/disagree	4 (3.9)
Somewhat disagree/neither agree nor disagree/somewhat agree	28 (27.2)
Strongly agree/agree	57 (55.3)
Prefer not to answer	2 (1.9)
Missing	**12**
3. Communication we receive from the department is open and honest. (n = 91)	
Strongly disagree/disagree	2 (1.9)
Somewhat disagree/neither agree nor disagree/somewhat agree	31 (30.1)
Strongly agree/agree	57 (55.3)
Prefer not to answer	1 (0.97)
Missing	**12**
4. People in the department care about me. (n = 91)	
Strongly disagree/disagree	3 (2.9)
Somewhat disagree/neither agree nor disagree/somewhat agree	38 (36.9)
Strongly agree/agree	50 (48.5)
Prefer not to answer	0 (0)
Missing	**12**
5. I feel welcome to express my true feelings at work. (n = 91)	
Strongly disagree/disagree	7 (6.8)
Somewhat disagree/neither agree nor disagree/somewhat agree	41 (39.8)
Strongly agree/agree	43 (41.7)
Prefer not to answer	0 (0)
Missing	**12**
6. People in the department who help the department achieve its strategic objectives are rewarded and recognized fairly. (n = 91)	
Strongly disagree/disagree	7 (6.8)
Somewhat disagree/neither agree nor disagree/somewhat agree	48 (46.6)
Strongly agree/agree	34 (33)
Prefer not to answer	2
Missing	**12**
7. Managers in the department are as diverse as the broader workforce. (n = 91)	
Strongly disagree/disagree	15 (14.6)
Somewhat disagree/neither agree nor disagree/somewhat agree	48 (46.6)
Strongly agree/agree	25 (24.3)
Prefer not to answer	3 (2.9)
Missing	**12**

#### Experiences with discrimination, harassment, and micro-aggressions

Over one-quarter (29%) of survey participants reported experiencing discrimination, harassment, or microaggressions (herein discrimination) within the organization, and 27% had considered leaving the department due to the work environment. Univariate regression analyses confirmed that those who had experienced discrimination were most likely to have considered leaving (OR = 12.03, 95% CI [2.79, 51.96]). No demographic factors were associated with considering leaving the department.

#### Dispute resolution and reporting

Approximately half (55%) of survey participants agreed they would feel comfortable objecting if they heard a discriminatory joke or statement and reporting inappropriate conduct if they witnessed (54%) or experienced it (49%). However, less than half (44%) reported that they would know where to go for help with inappropriate conduct, and 32% were confident that the hospital’s human resources (HR) department would respond appropriately if reported. Notably, 19% were not confident that the HR department would respond appropriately.

#### Recommended initiatives

Over 50% of participants indicated that all EDI activities listed were important/extremely important, however less than 30% agreed/strongly agreed they were being adequately addressed (Table 4). The greatest proportion of participants rated pay equity (78%), staff/learner diversity (76.9%), and counseling and support resources for microaggression, harassment or discrimination (72.2%) as very/extremely important. The top three EDI activities reported by the greatest number of participants as being adequately addressed were: increasing staff/learner diversity (32.1%), demonstrating commitment to inclusive excellence when hiring staff (26.7%), and formal staff mentorship (25%; Table 4).

**Table 4.** Adequacy and importance of EDI training and learning activities.

EDI Initiative	Adequacy* n (%)	Importance^#^ n (%)
Review pay equity	10 (14.5%)	62 (78%)
Increase the diversity of staff and learners	26 (32.1%)	60 (76.9%)
Counseling and other support resources for microaggression(s), harassment or discrimination	8 (11.4%)	57 (72.2%)
Examine departmental policies, processes, and practices using an equity lens	12 (15.6%)	56 (69.1%)
Provide implicit bias, equity, and anti-oppression training to all staff and learners	8 (10.4%)	56 (69.1%)
Increase awareness of equity, diversity, and inclusion challenges or experiences	18 (22.8%)	55 (69%)
Provide formal mentorship for staff and learners	20 (25%)	54 (68.4%)
Establish an equity, diversity, and inclusion community of practice	15 (19.2%)	51 (67.1%)
Provide ongoing learning opportunities to advance staff and learners’ EDI knowledge and skill	11 (14.3%)	54 (66.7%)
Include demonstrating commitment to advancing inclusive excellence as one of the criteria for hiring staff and learners	20 (26.7%)	54 (66.7%)
Improve recognition and/or compensation of service-related asks (e.g., committee work)	9 (12.3%)	52 (66%)
Include demonstrating a commitment to advancing inclusive excellence as one of the criteria for evaluation of staff and learners	16 (22.5%)	46 (59.7%)
Offer social events to foster social connection and community among staff and learners	17 (21.5%)	43 (54.4%)

*7-point Likert scale: 1 – inadequate to 7 – adequate. Only count and % for 6 – agree and 7 – strongly agree are reported.

^#^7-point Likert scale: 1 – not important at all to 7 – extremely important. Only count and % for 6 – very important and 7 – extremely important are reported.

### Staff interviews

Seventeen staff took part in semi-structured interviews, representing both clinical and research and three categories of staff (leadership, research/program coordination and administration). Approximately half identified as women (52.9%) and white (52.9%), and most (88%) were 30-60 years of age. For confidentiality reasons, no further demographic information is reported. Themes generated from interview data are organized based on perceived strengths that may help to facilitate departmental EDI advancement, and areas for improvement that may pose barriers to if unaddressed. Illustrative quotes are presented in Table 5.

**Table 5.** Key themes and illustrative quotes.

Themes	Illustrative Quotes
Facilitators	
Enthusiasm about EDI	*“I think the hospital could do a lot better… if I just think of [team x], there are things that are visibly good, and things that are visibly bad, or visibly opportunities to do better. I think there’s a want for inclusivity and representation and I think there’s opportunity to have the right people at the table to make sure that that happens.”* (Participant 8)
	*“Maybe one wish is for this to not just be a tokenism initiative, you know? So here we have two Black people, and also there is someone who used to live in India, and now I’m being really cruel here, but you know what I mean, because that’s what’s happening at the moment across organizations. You know, we’ve done our IDEA [inclusion, diversity, equity, and access], … let’s move on to the next big topic.”* (Participant 9)
Diversity	*“Maybe [job applicants] were a caregiver. Maybe they were, I don’t know, backpacking for a year… So things like that I try to be really mindful of… because I’m aware that our team is not super diverse.”* (Participant 1)
	*“I was just made aware recently of some departments that just don’t have women in leadership roles or people that are not White men. And if I think about our [team], [Leader] as our head female, and then three site leads, two are women and [leader] is the only man. So from like a gender perspective, I think we do well.”* (Participant 8)
	*“Our whole department is very female- we have a lot of females in our program. And it’s actually a really big issue, I think, for women in research… We are all well supported within our department, but outside of our department I don’t necessarily feel like we are. And so I think our department’s doing a good job.”* (Participant 10)
Openness to new ideas	*“I think there’s probably a lot we can improve upon, but … from my perspective, I think it’s a group that is open to change and would receive. I think it’s a group that wants information, wants to see things implemented, and wants to see change.”* (Participant 1)
	*“If a patient is upset with something or, you know, we recognize there is a gap. We are proactive and we are very open. And I think that’s key. We’re open to change in making things better, more inclusive, more equitable, more diverse.”* (Participant 2)
	*“I think it’s definitely improved. People are just more aware about… take pronouns for example, that was never something we did as the department, putting that in our [email] signature or learning how to appropriately address a patient and not assume pronouns… that was not something that was a focus, let alone something I actually saw in practice.”* (Participant 6)
Increased awareness of EDI	*“I think we’re probably more open to it because I think we’re more focused on the psychological, social, and cultural dimensions. So I think we’re more attuned to it probably than I presume we are.”* (Participant 13)
	*“So I had gone through life without having as much of an awareness of my privilege, right? [B]ecause I love all humans, and so I thought that was good enough, right? Like, I thought that was it, right? And so I didn’t care whether you were black or white, or whether you were gay or straight or whatever. But that’s not enough.”* (Participant 14)
	*“Having talked to others in the department, I think there’s a lot more attention to this area, you know, because of the Black Lives Matter movement, et cetera.”* (Participant 17)
Safe place for self-expression	*“I do feel like, at least for like [my team], I do feel like people do feel comfortable to be their authentic self, that they don’t have to put up a front, that if they do have concerns about either someone else, I think they do feel comfortable. That’s my opinion. I don’t have facts to support that obviously. … I haven’t really had people come to me and say, you know what, I feel this way because this person, you know, said something offensive to me. I personally haven’t experienced that.”* (Participant 2)
	*“Just even conflict resolution and if there’s been a problem or something that needed to be voiced in the department, I feel, based on my experience, personally and what I’ve noticed, it seems to be like a very safe space. A space where people feel free to voice their opinions and their thoughts.”* (Participant 6)
Areas for improvement	
Psychological safety	*“I wouldn’t be surprised if people said they didn’t feel comfortable bringing things forward. I don’t think we have a great language around it… I wouldn’t be surprised if people said they felt like they couldn’t be themselves.”* (Participant 1)
	*“[Physicians’] voices are louder than ours, in a sense of decisions. And if there is an element of disagreement or a difference of opinion, it’s not handled as respectfully and considerably as I would have liked it. It’s sort of the big man rules.”* (Participant 7)
	*“I think there is a level of fear, like, oh no, what am I saying? Am I going to get in trouble if I say this?”* (Participant 11)
	*“I think admins in particular don’t feel comfortable or safe raising their concerns. And I wish they would, and I’m always trying to tell people we work together. We don’t work in a hierarchy. I don’t know that the message gets across. So no, I don’t think they do [feel comfortable speaking up]. And I think the hierarchy stands. I think many [allied health professionals] are afraid to raise things to [physicians]. So it’s easy for me to say it doesn’t exist from where I sit, because I lead, right? I’m not afraid to speak up, but others are.”* (Participant 15)
Overwork and burn-out	*“It’s hard to get down to business when nothing is business as usual… I think the difficulty though right now is exacerbated by the fact that no aspect of [the hospital] feels like business as usual, and so it makes a difficult job even harder… Whenever I contact people, they’re always gone, on morale, right? … It’s an uphill battle.”* (Participant 3)
	*“So, for example, a couple of weeks ago, I think there was an application- I can’t remember the name of the acronym anymore. … I think it was to have students from visible minorities from high schools come and be able to try to do a kind of placement. And it’s like, okay, [to apply] you just need to read all these emails and fill out all this paperwork. And it’s like, oh my God. … I just don’t have time for this.”* (Participant 12)
	*“A lot of my work being in research is quantifiable, so one person might say I’m a better worker than other person and it’s very subjective. But sometimes… when you have like data in your hands, you know we’re doing better than whom. But I… really felt that maybe my English, maybe cultural differences, but there’s something about me that just makes it harder to move forward… I have really over the past 4 years a heavy heart. I had to start wondering… What is it about me that just makes it so difficult to move forward?”* (Participant 16)
Lack of diversity	*“We need to do a lot more of that [strategic hiring]. However, we don’t leave, and we don’t retire, so it’s not like there’s an abundance of hiring opportunities.”* (Participant 4)
	*“But, you know, we recently had… our last two new hires. I’m just like, well, you know, is it not time that we opened it up to a different gender? You know, let alone a different race? I feel like I love everybody that I work with. I think everybody is great. … I think they would learn more if… there were more differences on our team, you know.”* (Participant 5).
Cohesion, connection, and belonging	*“I think one challenge even with our own team is that we operate [out] of two physical sites. So even our team is not in one physical location. And that has posed some challenges amongst just connection and cohesion and bonding.”* (Participant 2)
	*“I think that, I would say number one is buy-in, which you have, but buy-in by [Leader] and [Manager] and really pushing that kind of on a higher level, like higher level meetings, but also team meetings within each [team] and really stressing the need for cohesion. I think that would help things out a lot… Sometimes it’s hard for people to really see the value in it unless it’s communicated elsewhere… in terms of bigger picture, what’s the benefits, what’s the value? I think those are things that need to really be pushed, almost to an extreme.”* (Participant 6)
	*“But there is a sense of, … I don’t know, pocketing, fragmentation, compartmentalization. You do you, I do me. And I don’t think it’s actually competitiveness. I thought for a while it’s competitiveness, but that’s kind of natural. Competition is always there, but I came more to the conclusion… it’s a very strong need for privateness and privacy and having a clear separation between what happens after work and what happens during work.”* (Participant 9)
Lack of communication and transparency	*“And so I think again, where possible [decision-making] should be bottom-up, right? Like people who are in the trenches, right? They’re going through it. They hear, see, and feel along with the patient. The solutions are there, you just have to elicit them. But I feel like a lot of times that is missed.”* (Participant 2)
	*“I think the spirit is there, it’s just that the policies and procedures have to catch up with it.”* (Participant 3)
Perceived lack of EDI knowledge	*“I think some of the other barriers are just lack of knowledge. And so feeling like people don’t do enough or don’t know enough to be able to make a meaningful difference or don’t know where to start in terms of educating themselves, or accessing appropriate resources.”* (Participant 4)
	*“I think sometimes when you’re in that corporate bubble, everyone in that bubble thinks information is accessible to all of [hospital]. And that’s not necessarily the case. It’s valuable that I had that experience [in a previous role] that now I know … it’s not people that I talk to or things that I know of now in my current role, which again, I think would be something to just make aware to staff, which would help the overall cause of improving EDI within the department. Because had I not had that experience, I would not know any of that.”* (Participant 6)

#### Facilitators of departmental EDI advancement

##### Enthusiastic about EDI

All interview participants viewed EDI as a core value and acknowledged the importance of advancing departmental EDI. An important facilitator identified in interviews was a collective enthusiasm for EDI and related initiatives. Nearly half discussed the importance of enthusiasm and curiosity around EDI for the success of EDI initiatives. In addition, while all participants felt the department was doing well in EDI compared to others, there was clear recognition that the department could and should be doing better.

##### Openness to new ideas

All participants described an open-minded and innovative department culture, where new ideas are encouraged and embraced, and staff are curious about new EDI efforts. This included an openness to EDI initiatives, willingness to experiment, and eagerness for ongoing education. Participants described feeling encouraged by leadership to voice new ideas and try new things, and commented on how staff were able to adapt to make departmental practices more equitable and inclusive. For example, one participant discussed how well the department incorporated and adapted to the sharing of gender pronouns in practice (e.g., not assuming people’s pronouns, adding pronouns to email signatures, etc.; Table 5).

##### Staff diversity

Gender diversity was named as a strength by several participants, who noted that there were more women in leadership positions than in many other departments in the hospital. While not necessarily captured by survey data, some participants felt that departmental diversity has improved over recent years, citing a broader eagerness to continue diversifying the department and specific efforts undertaken by staff. For example, one participant described efforts among their team to hire more clinicians from diverse backgrounds to reflect the hospital’s patient population. Another described endeavors to diversify hiring panels and attend to unconscious biases, including considering various reasons applicants may have gaps in their CVs (Table 5).

##### Increased awareness of EDI

Participants described an increased EDI-related awareness within the department. This is in part due to efforts individuals have undertaken on their own to expand their EDI knowledge, sparked by heightened awareness of health inequities through social movements such as Black Lives Matter (Table 4). Participants also reported an increased awareness of EDI through individual-level initiatives. Examples included participating in book clubs featuring underrepresented authors and seeking out training programs offered by other organizations. At the department level, participants spoke about the important work of several health equity-focused working groups, as well as the hiring of two co-leads to advance departmental EDI (Table 5).

##### Safe place for self-expression

Some participants described the department as a space where one can express their authentic self, thoughts, feelings, and experiences without overt discrimination from colleagues. Overall, these participants described the department as a place where people feel respected, accepted, and valued for who they are and what they bring to the workplace. However, it is notable that participants who described the department in this way were primarily in management or leadership positions. For example, one participant in leadership noted that, while they felt department staff do feel comfortable expressing themselves, no one had ever come to them with an issue (Table 5).

#### Possible barriers to departmental EDI advancement

##### Psychological safety

A critical barrier to advancing departmental EDI named by some participants was the lack of perceived psychological safety, defined as the belief that one can comfortably speak up (e.g., with dissenting opinions, concerns, mistakes, etc.) without fear of interpersonal humiliation or punishment.^20^ Some at the staff level reported feeling uncomfortable speaking up about issues or concerns at work, while, in contrast, those in leadership positions hoped that staff felt comfortable bringing issues forward. However, those in leadership also acknowledged that they were uncertain if staff were comfortable, as they had not received many explicit complaints indicating otherwise. Nearly half of interview participants drew attention to the department’s power dynamics, which was perceived as a barrier to creating a psychologically safe workplace. These power dynamics were described as a hierarchy with physicians at the top, therefore some participants felt uncomfortable opposing physicians. They believed this hierarchy contributes to the greater decision-making power and extensive access to resources afforded to physicians, who they felt are more valuable to the department. One participant indicated that they hoped department EDI initiatives would increase team members’ comfort in speaking up about issues and concerns regardless of position or discipline (Table 5).

##### Overwork and burnout

Several interview participants discussed feeling overworked and burnt-out, difficulties in maintaining a healthy work-life balance, and low morale. This was perceived to impact their capacity to participate in new EDI activities, implement new EDI initiatives, or take on new EDI responsibilities together with their existing workload (e.g., described as an “uphill battle”). Their limited capacity was perceived to be related in part to the long-term effects of COVID-19 on the department (Table 5). These feelings of overwork and burnout may be exacerbated among staff with marginalized identities or experiences. For example, a participant who reported that English was their second language wondered if language and cultural differences made it more difficult for them to advance within the department, despite evidence indicating they may be a good candidate for advancement.

##### Limited diversity

While gender diversity was emphasized as a strength of the department, participants voiced the need for greater diversity along other identity characteristics. Some highlighted the predominance of a white, heteronormative culture and stressed the need to increase diversity not only to better reflect the patient population, but to bring underrepresented knowledge, perspectives, and experiences into the department. With this, however, came a wariness of performativity and tokenistic initiatives. For example, one participant stressed the need for initiatives that move beyond diversity hiring quotas to make impactful changes that increase workplace inclusivity and belonging. However, others felt that increasing departmental diversity through strategic hiring may remain challenging given the perceived limited job opportunities (e.g., when current staff do not retire; Table 5).

##### Cohesion, connection, and collaboration

Participants felt a lack of cohesion, connection, and collaboration built into the department’s structure, both within and across teams. They described teams as “siloed” and separate from each other and highlighted a lack of cohesion between some disciplines within teams. For example, some felt there was a general lack of awareness of the activities of other teams, a lack of cohesion between medical doctors and allied health professionals within teams, and limited collaboration across teams on clinical programs and research projects. Some also described a perceived disconnect between leadership and frontline staff, including a lack of awareness from leadership regarding their EDI needs and experiences. Others described a perceived disconnect between colleagues due to factors including generational differences. Encouragingly, many expressed a desire to increase collaboration with their colleagues but felt that departmental culture was not conducive to collaboration. As a result, many participants emphasized the need for EDI training and interventions that encourage personal connection and team-building among colleagues to facilitate collaboration (Table 5).

##### Lack of communication and transparency

Interview participants highlighted the lack of communication and transparency as a barrier to successful implementation of departmental EDI initiatives. They discussed a desire for clarity from leadership and updated policies around EDI-related standards and responsibilities as staff. This included a need for greater transparency around decision-making, namely regarding the department’s Steering Committee. Participants wanted to be more involved in decision-making around EDI and desired bottom-up initiatives that accounted for the needs and experiences of frontline patient-facing staff. Related to this, participants expressed a lack of dedicated time and financial resources to enact meaningful, long-lasting EDI initiatives (Table 5).

##### Limited EDI knowledge

All participants described the need for further education and training around specific EDI concepts and how to find EDI-related resources on a day-to-day basis, while some described a lack of EDI knowledge among staff overall. Many were not able to recommend EDI resources, and some indicated that they did not know where to find EDI resources within the organization. In addition, participants raised the concern that some aspects of EDI, such as gender and racial diversity and overt forms of discrimination, were given more attention than other aspects of EDI, such as mental health, disability, and unconscious bias. They emphasized the need for further education on such neglected topics and the impact of power differentials at work. For example, one participant described taking part in EDI training that only superficially addressed workplace power differentials and felt that the topic should have been discussed in-depth given how these dynamics manifest in the workplace. In terms of EDI training, participants desired active, collaborative training opportunities that encourage personal connection, meaning-making opportunities, and provide a safe space for uncomfortable discussions (Table 5).

## Discussion

To our knowledge, this is one of only a handful of scientific reports assessing the EDI needs and experiences of healthcare staff at a healthcare institution. This study fills an important gap in the literature by increasing our understanding of the EDI needs and experiences of healthcare staff and barriers and facilitators to EDI advancement in healthcare. Overall, the quantitative and qualitative data are consistent and reflect the complexities of the EDI climate in the department under study. They demonstrate the value that healthcare staff placed on EDI and their eagerness to see the department continue its efforts in advancing inclusive excellence. The qualitative findings helped to contextualize and explain the results from the EDI climate survey, which contained contradictory findings. Notably, while over half of survey participants reported feeling respected, valued, and treated fairly within the department, less than half felt comfortable expressing their true thoughts and feelings at work and felt that people are rewarded and recognized fairly. While the department is in an advantageous position for EDI advancement, initiatives must foster inclusion, belonging, and psychological safety to maximize success.

Our findings demonstrate the negative impact of departmental power dynamics and hierarchies on the experience of psychological safety among employees. Our survey results indicated a lack of perceived psychological safety among participants. Interview participants elaborated on this further when they described feeling uncomfortable speaking up in opposition to those with more perceived power within the department, particularly physicians given their perceived higher status. For example, one participant in an administrative position reported not speaking up in opposition to physicians because they felt less valued and more replaceable. Others felt that physicians were more easily able to access resources and institutional support compared to other staff. These findings are in line with prior research examining power hierarchies in healthcare, highlighting their detrimental effects on workplace culture by dictating who has the power and resources.^8^ Also integral to this discussion are consistent sociodemographic disparities in medicine,^6,7^ which beg an important question – if physicians are positioned at the apex of healthcare hierarchies, and equity-seeking groups remain underrepresented in these positions, *who* most often has the power to speak in healthcare workplaces? The answer is often white cisgender heterosexual men. Diversity and training in psychological safety is critical for leadership to support EDI advancement.^7^ Psychological safety is a significant yet often overlooked component of EDI advancement – its absence can create a toxic culture whereby those with less power and status, regarding occupation/position and sociodemographics, are reluctant to speak up in fear of repercussions.^20^

While participants perceived departmental gender diversity as a strength, only 28% of survey participants agreed that management was as diverse as the broader workforce. Interview participants elaborated on several axes of identity that were not sufficiently represented (e.g., race, country of origin, sexual orientation, gender identity). They felt this was problematic given the diversity of the patient population served by the hospital. To improve the diversity of the department’s workforce, several participants recommended improving hiring processes, including removing barriers for applicants who may not have traditional resumes and may have ordinarily slipped through the cracks. However, attention to hiring practices and inviting diverse perspectives must come with attention to psychological safety. While the commonly-held belief is that increased diversity results in increased performance, the opposite is often true – teams that are more diverse may clash due to communication hiccups when people from different backgrounds work together.^20^ The key to ensuring that greater diversity contributes to increased performance and job satisfaction is psychological safety. Therefore, it would be insufficient to increase diversity without also ensuring department culture values expression of different viewpoints and seeks to build shared ownership over success.

Cohesion, collaboration, and belonging, both among staff and between staff and leadership, was another important finding in this study. Most staff felt their ideas and suggestions were fairly considered and that colleagues respected and valued each other’s opinions. However, less reported feeling cared for by colleagues and feeling they belonged in the department. Indeed, several interview participants reported feeling disconnected from department colleagues both physically (as staff are located in different buildings) and emotionally (partly due to a lack of collaboration). This finding emphasizes the difference between diversity (being invited to the metaphorical dance) and belonging (being asked to dance). That is, while ensuring a diversity of perspectives by bringing a range of people into the workplace is important, ensuring that everyone feels they *belong* and can actively bring their differences to build a welcoming culture is a measure of a truly inclusive workplace.^20^

A key component of fostering belonging is ensuring positive relationships are built between staff and leadership through clear, transparent communication.^20^ While 63% of survey respondents agreed that communication from the department is open and honest, some interview participants expressed a desire for more transparent communication from leadership around decision-making and EDI-related standards. While participants were generally knowledgeable about different aspects of EDI, even those who were further along in their EDI learning journeys (e.g., active, advocacy) reported not knowing of specific EDI-related policies and procedures that were available for guidance. This led to some participants feeling unsure of who or where to turn to for EDI-related issues, which was reflected in the low proportion of survey respondents who were comfortable reporting discrimination, knew where to go for help, and trusted hospital HR to respond appropriately. It appears there is an overall lack of clarity around EDI policies and expectations in part because these are not consistently communicated by department leadership. This in turn could impact staff’s perceived trust in the organization to respond adequately to EDI-related issues. To ensure all staff are aware of EDI-related policies and guidelines, it is important to develop a communication strategy to inform staff of EDI resources (e.g., in team meetings/huddles to ensure it is not overlooked in staff-wide email notifications).

Our findings also showed a desire for EDI initiatives that would allow staff to connect across the department to facilitate greater cohesion and collaboration. However, greater involvement in such initiatives would first require attending to the high levels of burnout among healthcare providers at large, which worsened during the COVID-19 pandemic and subsequent recovery periods, which have been rife with staffing shortages amidst pressure to meet demands on the healthcare system.^21^ Feeling burnt out at work was endorsed by approximately 24% of survey participants, which is within the range of burnout prevalence rates among various health professions (e.g., 8-73%).^21,22^ Interview participants provided further context for this finding, with one citing that “no aspect of [the hospital] feels like business as usual” due to COVID-19 and ensuing systemic and institutional pressures, which significantly impacted staff morale (Table 5). Lack of morale due in part to various stressors has depleted the time and mental resources required to engage in new initiatives and has negatively impacted overall attitudes and workplace culture. Indeed, burnout has been found to be a potential trigger for instances of workplace incivility, or behaviours considered rude or disrespectful, and overall show a disregard for norms around respectful and ethical conduct between colleagues.^23^ While these instances are not as overtly aggressive as assault or bullying, they can impact workplace culture by increasing levels of anxiety, distress, exhaustion and fatigue, and burnout, along with decreasing psychological safety, well-being, productivity, and work engagement.^24^

Of concern, 29% of survey respondents indicated that they had experienced some form of discrimination in the workplace. This question (modelled after the question presented in the McMaster University Diversity and Climate Survey^15^) included a potential range of experiences, from comments or actions that constitute microaggressions (e.g., comments such as “You speak English so well”), to those that constitute harassment (e.g., yelling or shouting which intimidates another person, unwanted sexual comments), or discrimination (e.g., not taking applications from job seekers of a certain racial/ethnic background). Regardless of prevalence, the impact of these occurrences is not insignificant, as those who had experienced discrimination were significantly more likely to have considered leaving the department. This is in line with other research that shows a relationship between workplace bullying and increased employee turnover adversely affecting employees’ physical and mental health, and quality of patient care.^7^

### Limitations

This study has certain limitations. First, we assessed the EDI-related needs and experiences of staff and learners in one department of a single hospital shortly after an organizational diversity climate survey was implemented. Sampling for participant interviews was purposive rather than using convenience sampling, to ensure representation from across teams, disciplines, and levels of institutional power (e.g., staff, middle management, leadership). Only department staff were recruited to participate in qualitative interviews, therefore learner experiences were not captured in this component of the study. A notable limitation of the climate survey was the use of measurement scales that have not yet been validated. Despite these limitations, a response rate of approximately 50% was achieved, which is higher than other published surveys of healthcare providers in the same hospital network where the response rate was approximately 29%,^25^ and surveys at other institutions.^26^

### Recommended solutions and implications for research

To date, the workplace EDI literature has focused primarily on aspects of compositional diversity, while studies examining the role of inclusion are limited.^27^ While diversity reflects differences in a group’s compositional makeup, inclusion refers more explicitly to feelings of belonging, dignity, empowerment, and meaningful engagement within a community.^5^ Our findings emphasize the need for greater attention to workplace inclusion, as many of the areas identified as potential barriers to EDI advancement were related to inclusion and belonging. Therefore, we recommend that future workplace EDI research and initiatives increase their focus on inclusion. The inclusion framework by Shore and colleagues^28^ aligns with the EDI needs expressed by healthcare staff in this study. It offers a practical approach for achieving a workplace with strong perceived inclusiveness in the overall climate (e.g., valuing diversity, treating all employees fairly), leadership (e.g., leader behaviours that reflect EDI values), and practices (e.g., those that promote cohesion and belonging) that contribute to greater job satisfaction, staff retention, and workplace relationships.

To enact meaningful and lasting changes that advance workplace EDI, both individual and organizational change is required. The issues identified in this departmental needs assessment require a multi-faceted, multi-level approach beginning with increasing the EDI knowledge, skill and self-efficacy of staff in a manner that builds cohesion and belonging, enhances psychological safety, and acknowledges feelings of overwork and burn-out. This must occur in tandem with initiating policies and procedures to attract, support and retain a diverse workforce, and respond to experiences of microaggression, discrimination and harassment. Drawing on Shore and colleagues’ inclusion framework,^28^ adult learning theory,^29^ and based on the findings of this needs assessment, we developed and are in the process of evaluating an EDI Story Huddle Learning Series (SHLS). The SHLS is focused on increasing staff knowledge and self-efficacy to address unconscious bias, microaggressions, power differentials, and psychological safety. Given the perceived lack of and desire for cohesion and belonging in this department, team-based learning activities were identified as the most appropriate pedagogical approach. Hence, in the SHLS, a trained EDI facilitator presented staff/learners with complex case scenarios that were explored with guiding questions in small-group huddles, followed by a discussion as a larger group. In parallel, we have undertaken an EDI-informed review of departmental hiring policies and procedures and are developing an equitable hiring practices toolkit to attract, support, and retain a diverse workforce. Importantly, we plan to conduct ongoing evaluations to assess the extent to which these initiatives and future planned initiatives result in transformative systems-level change.

Because this study was conducted in one department of a single institution, we caution against generalizing these findings given that EDI-related knowledge, buy-in, and needs may differ across workplaces. We instead recommend that those wishing to make changes based on these findings begin by conducting EDI needs assessments in their own insitutions. We hope this paper serves as a useful methodological example of how to conduct EDI needs assessments in a healthcare organization, questions to ask, and potential findings to explore. Developing intitiaves to advance inclusive excellence based on institutional needs assessments will allow for more relevant and tailored approaches to advance EDI in healthcare workplaces. To advance the science and practice of EDI, future research should devise how to map EDI needs to evidence-based strategies to optimize the long-term effectiveness and impact of EDI initiatives.

## Conclusions

This needs assessment revealed that EDI is a core value of the department under study, and there is a collective belief that the department could and should do more to advance EDI and inclusive excellence. We found there to be strengths present within the department that could help facilitate EDI advancement, including a growing awareness of and enthusiasm for EDI learning and a commitment to promoting staff diversity. While these strengths put the department in an advantageous position to pursue inclusive excellence, we also identified critical areas for improvement that may serve as barriers to EDI advancement. These barriers reflect areas where EDI values of staff have not been effectively translated into specific behaviours, policies, and procedures. Our findings underscore the importance of meeting compositional diversity with tailored policies and initiatives that contribute to true inclusion and belonging in healthcare workplaces broadly. Such environments would ensure staff feel they belong, are psychologically safe to express themselves, feel connected to colleagues, and are encouraged to maintain a healthy work-life balance. This, of course, requires simultaneous organizational action to tangibly change institutional structures and procedures using tailored initiatives to maximize successful uptake and implementation.

## Supplemental Material

Supplemental Material - Examining healthcare staff views and experiences with equity, diversity, and inclusion (EDI) in a multi-disciplinary healthcare setting: A mixed methods needs assessment to advance inclusive excellence Supplemental Material for Examining healthcare staff views and experiences with equity, diversity, and inclusion (EDI) in a multi-disciplinary healthcare setting: A mixed methods needs assessment to advance inclusive excellence by Lauren Rochelle Squires, Logan Meyers, Myann Marks, Eryn Tong, Ekaterina An, Camilla Zimmermann, and Jacqueline L Bender in Health Services Management Research

## Data Availability

The datasets generated and analyzed during the current study are not available given that participants may be identifiable, but are available from the corresponding author upon reasonable request.*
