# Supplementary material for: Examining healthcare staff views and experiences with equity, diversity, and inclusion (EDI) in a multi-disciplinary healthcare setting: A mixed methods needs assessment to advance inclusive excellence
Source: Health Serv Manage Res. 2025 Oct 9;39(2):66–81. doi: 10.1177/09514848251387042 (PMC13062439; doi:10.1177/09514848251387042)
Supplement: Supplemental Material - Examining healthcare staff views and experiences with equity, diversity, and inclusion (EDI) in a multi-disciplinary healthcare setting: A mixed methods needs assessment to advance inclusive excellence [file sj-pdf-1-hsm-10.1177_09514848251387042.pdf]

Appendix S1

Good Reporting of A Mixed Methods Study (GRAMMS)	
Guidelines	Page number
1. Describe the justification for using a mixed methods approach to the research question.	Page 5
2. Describe the design in terms of the purpose, priority and sequence of methods.	Page 5
3. Describe each method in terms of sampling, data collection and analysis.	Page 5
4. Describe where integration has occurred, how it has occurred and who has participated in it.	Page 7
5. Describe any limitation of one method associated with the present of the other method.	Page 17
6. Describe any insights gained from mixing or integrating methods	Page 14

Appendix S2: EDI Climate Survey

Section 1: Eligibility

1. Do you currently work in the [department] as a staff member and/or learner?
    - o Yes
    - o No

Section 2: Demographics

2. What is your current age?
    - o Under 20 years
    - o 20 to 29 years
    - o 30 to 39 years
    - o 40 to 49 years
    - o 50 to 59 years
    - o 60 to 69 years
    - o 70 years and over
    - o Prefer not to answer
3. What is your gender identity? Select all that apply.
    - o Agender
    - o Bigender
    - o Gender fluid
    - o Man
    - o Non-binary
    - o Non-conforming
    - o Pan-gender
    - o Questioning or unsure
    - o Transgender man
    - o Transgender woman
    - o Two-Spirit
    - o Woman
    - o Not listed (specify): _____________
    - o Do not know
    - o Prefer not to answer
4. What is your sexual orientation? Select all that apply.
    - o Asexual
    - o Bisexual
    - o Gay
    - o Lesbian
    - o Pansexual
    - o Queer
    - o Questioning or unsure
    - o Same-gender loving
    - o Straight (heterosexual)
    - o Two-Spirit

- Not listed (specify): _________
- Do not know
- Prefer not to answer

5. Do you identify as an Indigenous person? Select all that apply.
    - Yes, First Nations
    - Yes, Métis
    - Yes, Inuk/Inuit
    - Yes, another Indigenous identity (specify): ___________
    - No
    - Do not know
    - Prefer not to answer
6. What category(ies) best describes your race or racial background? Select all that apply.
    - Black (e.g., African, African Canadian, Afro-Caribbean descent)
    - East Asian (e.g., Chinese, Japanese, Korean, Taiwanese descent)
    - Indigenous (e.g., First Nations, Métis, Inuk/Inuit)
    - Latin American (e.g., Hispanic or Latin American descent)
    - Middle Eastern (e.g., Arab, Persian, West Asian descent (e.g., Afghan, Egyptian, Iranian, Kurdish, Lebanese, Turkish))
    - South Asian (e.g., South Asian descent (e.g., Bangladeshi, Indian, Indo-Caribbean, Pakistani, Sri Lankan))
    - Southeast Asian (e.g., Cambodian, Filipino, Thai, Vietnamese, or other Southeast Asian descent)
    - White (e.g., European descent)
    - Multiracial/ethnic (with at least 1 parent in a non-White group above)
    - Another race category (specify): ___________
    - Do not know
    - Prefer not to answer
7. What is your religious or spiritual affiliation? Select all that apply.
    - Agnosticism
    - Animism or Shamanism
    - Atheism
    - Baha'i Faith
    - Buddhism
    - Christian Orthodox
    - Christian
    - Confucianism
    - Hinduism
    - Islam
    - Jainism
    - Judaism
    - Native Spirituality
    - Pagan
    - Protestant

- Rastafarianism
- Roman Catholic
- Sikhism
- Spiritual
- Unitarianism
- Zoroastrianism
- Not listed (specify): _________
- Not Applicable, I do not have a religious or spiritual affiliation
- Do not know
- Prefer not to answer

8. Do you identify as a person with a disability and/or impairment? Select all that apply.
   - No, I do not have a disability or impairment.
   - Yes, I have a neurodevelopmental disorder, intellectual, cognitive, and/or learning disability and/or impairment (e.g., Autism, ADHD, etc.)
   - Yes, I have a physical disability and/or impairment (e.g., mobility, dexterity, etc.)
   - Yes, I have a sensory disability and/or impairment (e.g., deaf, deafened, hard of hearing, vision loss, blind, etc.)
   - Yes, I have a mental health illness and/or impairment (e.g., schizophrenia, bipolar, generalized anxiety disorder, depression, etc.)
   - Yes, I have another disability and/or impairment (e.g., chronic medical condition, speech impairment, etc.)
   - Do not know
   - Prefer not to answer
9. Have you requested accommodation(s) at work for any limitation(s) or disability(ies)? Select all that apply.
   - Yes, I have requested accommodation and all my disability-related needs have been accommodated
   - Yes, I have requested accommodation and some of my disability-related needs have been accommodated
   - Yes, I have requested accommodation and none of my disability-related needs have been accommodated
   - No, I have not requested accommodation for my disability-related needs
   - No, I have not requested accommodation for my disability-related needs that could be accommodated
   - I do not require accommodation
   - Prefer not to answer
10. Do you provide care to any dependents (including but not limited to a child, parent, or other family member/relative, person with a disability, etc.) for whom you are responsible for their care on either a full-time or part-time basis?
    - Yes, full-time
    - Yes, part-time
    - No
    - Prefer not to answer

11. What is the highest level of education of your most highly educated parent or guardian?
    - o Less than high school
    - o Graduated high school
    - o Attended college/CEGEP but did not earn a certificate, diploma, or degree
    - o Attended university but did not earn a degree
    - o Completed a college/CEGEP certificate or diploma
    - o Bachelor's degree (e.g., BA, BSc, etc.)
    - o Professional degree (e.g., medicine, law, pharmacy, dentistry, etc.)
    - o Master's degree
    - o Doctoral degree
    - o Skilled trades
    - o Another category; may include non-traditional education from outside Canada (specify): _______________
    - o Do not know
    - o Prefer not to answer
12. Were you born in Canada?
    - o Yes
    - o No
    - o Do not know
    - o Prefer not to answer
13. What is your position in the [department]? Select all that apply.
    - o Staff
    - o Learner (e.g., paid, or unpaid clinical and research students in a degree or diploma program)
    - o Prefer not to answer

Section 3: EDI Knowledge

14. Please rate your agreement with the following statements:								
	1- Strongly disagree	2- Disagree	3- Somewhat disagree	4- Neither agree nor disagree	5- Somewhat agree	6- Agree	7- Strongly agree	Prefer not to answer
I believe equity, diversity, and inclusion should be a leading priority for the [department].								
I have working knowledge of anti-								

oppression								
I have working knowledge of cultural safety								
I have working knowledge of cultural safety								
I regularly apply equity, diversity and inclusion principles in my work/learning/ teaching								

15. How would you describe where you are on the EDI learning journey?
    - Unaware – Unaware of the impact EDI can have on people and work
    - Discovery – Interested in learning about the issues and barriers that stand in the way of EDI
    - Active – Advancing knowledge, engaging in tough conversations, and actively applying EDI principles in my work
    - Advocacy – Championing the advancement and success of others including those marginalized by gender, Indigenous Peoples, racialized minorities, persons with disabilities, and members of LGBTQ2S+ communities
    - Other (specify): ___________
    - Prefer not to answer
16. How would you describe where the majority (e.g., more than 50%) of your [department] colleagues are on their EDI learning journey?
    - Unaware – Unaware of the impact EDI can have on people and work
    - Discovery – Interested in learning about the issues and barriers that stand in the way of EDI
    - Active – Advancing knowledge, engaging in tough conversations, and actively applying EDI principles in my work
    - Advocacy – Championing the advancement and success of others including those marginalized by gender, Indigenous Peoples, racialized minorities, persons with disabilities, and members of LGBTQ2S+ communities
    - Other (specify): ___________
    - Prefer not to answer

Section 4: EDI Climate

17. Overall, how would you rate the EDI climate within the following areas?								
	1- Extremely	2-Very poor	3-Poor	4-Fair	5- Good	6-Very good	7- Excellent	Prefer not to

	poor							answer
Your [team]								
[Department]								
[Hospital]								

18. Overall, do you feel that the EDI climate is improving, staying the same or getting worse in the following areas:				
	Improving	Staying the same	Getting worse	Prefer not to answer
Your [team]				
[Department]				
[Hospital]				

19. In your primary role within the [department], please indicate the extent to which you agree or disagree with the following statements:								
	1- Strongly disagree	2- Disagree	3- Somewhat disagree	4-Neither agree nor disagree	5- Somewhat agree	6-Agree	7- Strongly agree	Prefer not to answer
I feel valued as an individual within the [department]								
I feel I have to work harder than I believe my colleagues do to achieve the same recognition								
My colleagues and coworkers in the [department] treat me with dignity and respect								
It is difficult to ask my colleagues and coworkers for help								
I feel like a part of the [department]								
I am reluctant to bring up concerns or issues for fear that it will affect my performance evaluation								

I have sufficient opportunities for advancement within the [department]								
I feel burdened by service responsibilities (e.g., committee work) beyond those of my colleagues								
I have received support for advancement within the [department]								
I feel burned out by my work								
Leadership in the [department] demonstrates a commitment to equity, diversity, and inclusion								

20. Please indicate the extent to which you agree or disagree with the following statements:								
	1-Strongly disagree	2-Disagree	3-Somewhat disagree	4-Neither agree nor disagree	5-Somewhat agree	6-Agree	7-Strongly agree	Prefer not to answer
People in the [department] who help the department achieve its strategic objectives are rewarded and recognized fairly								
People in the [department] respect and value each other's opinions								
Members of my team fairly consider ideas and suggestions offered by other								

team members								
I feel welcome to express my true feelings at work								
Communication we receive from the [department] is open and honest								
People in the [department] care about me								
Managers at the [department] are as diverse as the broader workforce								

21. Have you ever considered leaving the [department] because of the work environment? Choose one of the following answers.
    - o Yes (please elaborate): ____________
    - o No
    - o Prefer not to answer

22. How equitable do you feel the following practices or processes have been in the [department] with respect to member's sociodemographic characteristics (e.g., age, sex, gender, sexual orientation, race, disabilities, income, etc.)?								
	1-Not at all equitable	2	3	4- Moderately equitable	5	6	7- Extremely equitable	Prefer not to answer
Recruitment policies and practices								
Annual review								
Promotion decisions								
Salary decisions								
Allocation of resources (i.e., space, equipment, etc.)								
Growth opportunities (e.g., opportunities to acquire new knowledge and skills)								
Mentorship								

(e.g., support from a colleague who provides direct guidance, advice, feedback on skills and coaching for career development or advancement)								
Sponsorship (e.g., support from a colleague in a position of power who speaks on your behalf in an effort to promote you)								
Leadership opportunities (e.g., opportunities to lead a project, train/mentor staff, etc.)								

23. If you would like to elaborate on any of your responses above, please comment here: __________________

Section 5: Experiences with Microaggression, Discrimination, and Harassment

24. Have you ever experienced microaggressions, discrimination or harassment within the [department]? Choose one of the following answers:
    - o Yes
    - o No
    - o Unsure
    - o Prefer not to answer
25. Who was the source of conduct related to your experiences of microaggressions, discrimination or harassment within the [department]? Select all that apply:
    - o [Department] colleagues
    - o Patients or family members
    - o Other hospital staff (not members of the [department])
    - o Prefer not to answer
    - o Other (specify): _______________

26. If there is any other information that you would like to provide about your personal experiences with microaggressions, discrimination or harassment within the [department], please comment here: ____________

27. Please indicate the extent to which you agree or disagree with the following statements related to dispute resolution and reporting within the [department]:

	1- Strongly disagree	2- Disagree	3- Somewhat disagree	4-Neither agree nor disagree	5- Somewhat agree	6-Agree	7- Strongly agree	Prefer not to answer
If I were to hear members of the [department] make discriminatory jokes or statements, I would feel comfortable openly expressing my distaste/objection								
If I or a colleague were subject to microaggressions, exclusionary, intimidating, offensive, and/or hostile conduct, or disparaging remarks within the [department], I would know where to go for help								
If I were to experience microaggressions, exclusionary, intimidating, offensive, and/or hostile conduct, or disparaging remarks within the [department], I would feel comfortable reporting it to a faculty, staff or administrator								
If I were to								

witness microaggressions, exclusionary, intimidating, offensive, and/or hostile conduct, or disparaging remarks within the [department], I would feel comfortable reporting it to a faculty, staff or administrator								
If I were to report harassment or discrimination at [department], I am confident that [hospital]'s People and Culture (e.g., HR) would respond to it appropriately								

Section 6: Initiatives to Advance Inclusive Excellence

28. Have you participated in any EDI training or learning activities?
    - o Yes
    - o No
    - o Unsure
    - o Prefer not to answer

    If yes, please provide the name of the learning activity and the organization that offered it: _____________

29. Are the following EDI initiatives being adequately addressed within the [department]?

	1-Inadequate	2	3	4-Neither adequate nor inadequate	5	6	7-Adequate	Prefer not to answer
Provide implicit bias, equity, and anti-oppression training to all staff and learners								
Provide ongoing learning opportunities to advance staff and learners EDI knowledge and skill								
Increase the diversity of								

staff and learners								
Include demonstrating commitment to advancing inclusive excellence as one of the criteria for hiring of staff and learners								
Include demonstrating commitment to advancing inclusive excellence as one of the criteria for evaluation of staff and learners								
Increase awareness of equity, diversity and inclusion challenges or experiences								
Provide formal mentorship for staff and learners								
Provide, promote, and improve access to counselling and other support resources (e.g., medical, legal, workplace accommodations) for people who have experienced microaggression(s), harassment or discrimination								
Examine departmental policies, processes, and practices using an equity lens								
Review pay equity								
Improve recognition and/or compensation of service-related asks (e.g., committee work)								
Offer social events to foster social connection and community among staff and learners								
Establish an equity, diversity and inclusion community of practice								

30. How important are the following initiatives to improving the inclusive environment of the [department]?

	1-Inadequate	2	3	4-Neither adequate nor inadequate	5	6	7-Adequate	Prefer not to answer
Provide implicit bias, equity, and anti-oppression training to all staff and learners								
Provide ongoing learning opportunities to advance staff and learners EDI knowledge and skill								
Increase the diversity of staff and learners								
Include demonstrating commitment to advancing inclusive excellence as one of the criteria for hiring of staff and learners								
Include demonstrating commitment to advancing inclusive excellence as one of the criteria for evaluation of staff and learners								
Increase awareness of equity, diversity and inclusion challenges or experiences								
Provide formal mentorship for staff and learners								
Provide, promote, and improve access to counselling and other support resources (e.g., medical, legal, workplace accommodations) for people who have experienced microaggression(s), harassment or discrimination								
Examine departmental policies, processes, and practices using an equity lens								
Review pay equity								
Improve recognition and/or compensation of service-related asks (e.g., committee work)								
Offer social events to foster social connection and community among staff and learners								
Establish an equity, diversity								

and inclusion community of practice								

Are there any other initiatives that you recommend to improve the inclusive environment of the [department]?

31. Please share any other thoughts, comments or suggestions you may have about the topics covered in this survey, including anything that would contribute to advancing inclusive excellence in the [department].

32. Please let us know if there are any important questions related to climate or demographic information that were not included in the survey.
